# Investigation of independent reinforcement learning algorithms in multi-agent environments

**DOI:** 10.3389/frai.2022.805823

**Published:** 2022-09-20

**Authors:** Ken Ming Lee, Sriram Ganapathi Subramanian, Mark Crowley

**Affiliations:** Department of Electrical and Computer Engineering, University of Waterloo, Waterloo, ON, Canada

**Keywords:** multi-agent reinforcement learning, reinforcement learning, deep learning, machine learning, artificial intelligence

## Abstract

Independent reinforcement learning algorithms have no theoretical guarantees for finding the best policy in multi-agent settings. However, in practice, prior works have reported good performance with independent algorithms in some domains and bad performance in others. Moreover, a comprehensive study of the strengths and weaknesses of independent algorithms is lacking in the literature. In this paper, we carry out an empirical comparison of the performance of independent algorithms on seven PettingZoo environments that span the three main categories of multi-agent environments, i.e., cooperative, competitive, and mixed. For the cooperative setting, we show that independent algorithms can perform on par with multi-agent algorithms in fully-observable environments, while adding recurrence improves the learning of independent algorithms in partially-observable environments. In the competitive setting, independent algorithms can perform on par or better than multi-agent algorithms, even in more challenging environments. We also show that agents trained *via* independent algorithms learn to perform well individually, but fail to learn to cooperate with allies and compete with enemies in mixed environments.

## 1. Introduction

One of the simplest ways to apply reinforcement learning in multi-agent settings is to assume that all agents are independent of each other. In other words, every other agent is seen as part of the environment from any agent's perspective. Independent algorithms (i.e., single-agent algorithms) face the issue of non-stationarity in the multi-agent domain due to the violation of the Markovian property in a Markov Decision Process (Choi et al., 1999). As a result, independent algorithms lack convergence guarantees, and are not theoretically sound in the multi-agent setting (Tan, 1993). Despite these shortcomings, independent algorithms have the advantage of requiring lower computational resources and being easier to scale to large environments than traditional multi-agent algorithms which perform exact opponent modeling of each agent. In practice, prior works have reported mixed performance for independent algorithms in different multi-agent domains (Shoham and Leyton-Brown, 2008; Zawadzki et al., 2014; Lowe et al., 2017; Tampuu et al., 2017; Foerster et al., 2018; Rashid et al., 2018; Berner et al., 2019). However, a study of the strengths and weaknesses of independent algorithms across various categories within the multi-agent domain is lacking in the literature.

In this paper, we investigate the empirical performance of independent algorithms in multi-agent settings, and compare them to various multi-agent algorithms under the Centralized Training and Decentralized Execution scheme (Oliehoek et al., 2008; Kraemer and Banerjee, 2016). We evaluate these algorithms on 7 multi-agent environments from the PettingZoo library (Terry et al., 2020b), which span the 3 main categories of multi-agent environments (i.e., cooperative, competitive, and mixed) (Busoniu et al., 2008; Canese et al., 2021; Zhang et al., 2021; Gronauer and Diepold, 2022). We show that independent algorithms can perform on par with multi-agent algorithms in the cooperative, fully-observable setting, and adding recurrence allows them to perform well compared to multi-agent algorithms in partially observable environments. In the competitive setting, we show that parameter sharing alongside the addition of agent indicators allow independent algorithms to perform on par or better than multi-agent algorithms, even in challenging environments. For the mixed setting, we show that agents of independent algorithms learn to perform well individually, but fail in learning to cooperate with allies and compete against enemies.

## 2. Background information

In this section, we provide readers with a brief overview of the various concepts and algorithms that are used throughout the paper.

### 2.1. Reinforcement learning

In Reinforcement Learning (RL), an agent interacts with the environment by making sequential decisions (Sutton and Barto, 2018). At every time step, denoted as *t*, the agent observes a state *s*_*t*_ from the environment, and takes an action *u*_*t*_. This action is executed in the environment, which returns a reward *r*_*t*_ and the next state *s*_*t*+1_ that are determined by the reward function *R*(*s*_*t*_, *u*_*t*_) and the transition probability, *P*(*s*_*t*+1_|*s*_*t*_, *u*_*t*_), respectively. Critically, *R*(*s*_*t*_, *u*_*t*_) and *P*(*s*_*t*+1_|*s*_*t*_, *u*_*t*_) are part of the environment, and are usually unknown to the agent. Since the transition probability *P*(*s*_*t*+1_|*s*_*t*_, *u*_*t*_) conditions the next state *s*_*t*+1_ purely on the current state *s*_*t*_ and action *u*_*t*_, it satisfies the Markov property (Markov, 1954). This interaction between the agent and the environment is called a Markov Decision Process (MDP) (Bellman, 1957). The objective of an RL agent is to learn a policy π(*u*_*t*_|*s*_*t*_), which maps a state to an action that maximizes the expected cumulative reward it receives from the environment. This is written as $\underset{t}{\sum }\gamma^{t}r_{t}$, where γ∈[0, 1) represents a discount factor on future rewards.

### 2.2. Multi-agent reinforcement learning

The single-agent MDP framework is extended to the Multi-Agent Reinforcement Learning (MARL) setting in the form of stochastic games (Shapley, 1953). In an *N*-agent stochastic game, at every time step, each of the *n* agents, identified by *j*∈{1, 2, …, *n*} across all agents, takes an action $u_{t}^{j}$. The joint action is written as $u_{t}≜\langle u_{t}^{1},\ldots ,u_{t}^{N}\rangle$. Every agent receives an agent specific reward through the reward function *R*(*s*_*t*_, ***u*_*t*_**, *j*). State transitions of the environment are determined by the transition probability *P*(*s*_*t*+1_|*s*_*t*_, ***u*_*t*_**), which conditions on the state and the joint action at timestep *t*.

### 2.3. Centralized training and decentralized execution

While it is technically possible to learn a centralized controller that maps a state to a distribution over the joint action space, the number of possible combinations of actions grows exponentially with the number of agents. This makes centralized control intractable for environments with many agents. Therefore, this paper is mainly focused on multi-agent algorithms which correspond to a Centralized Training and Decentralized Execution (CTDE) scheme (Oliehoek et al., 2008; Kraemer and Banerjee, 2016). A CTDE algorithm has two phases. During the control phase, where policies are deployed in the environment, rather than using a centralized controller to take actions for all agents, decentralized agents make decisions based on their individual observations. During the prediction phase, centralized training is performed, which allows for extra information (e.g., the state) to be utilized, as long as this is not required during the control phase.

### 2.4. Cooperative, competitive, and mixed

This paper follows the convention of classifying every multi-agent algorithm and environment studied into one of three categories—cooperative, competitive, and mixed (cooperative-competitive) (Busoniu et al., 2008; Canese et al., 2021; Zhang et al., 2021; Gronauer and Diepold, 2022).

In the cooperative setting, agents collaborate with each other to achieve a common goal. As a result, it is very common for all agents to share the same reward function (i.e., a team goal) (Chang et al., 2003). Also known as the multi-agent credit assignment problem, every agent has to deduce its own contributions from the team reward (Chang et al., 2003). Algorithms studied in this paper that explicitly address the multi-agent credit-assignment problem include an on-policy algorithm Counterfactual Multi-Agent Policy Gradients (COMA) (Foerster et al., 2018), and an off-policy algorithm, QMIX (Rashid et al., 2018), which addresses the poor sample efficiency of on-policy algorithms. Additionally, the CommNet (Sukhbaatar et al., 2016) extension on top of COMA is utilized for specific cooperative environments to promote communication between cooperative agents. Other multi-agent algorithms that are considered for the cooperative scenario include multi-agent variants of single-agent algorithms, such as Multi-Agent Deep Deterministic Policy Gradient (MADDPG) (Lowe et al., 2017) and Multi-Agent Proximal Policy Optimization (MAPPO) (Yu et al., 2021).

In the competitive setting, agents play a zero-sum game, where an agent's gain is another agent's loss. In other words, $\underset{a}{\sum }r\left(s,u,a\right)=0\forall s,u$. Algorithms that are studied specifically in this paper include Deep Reinforcement Opponent Network (DRON) (He et al., 2016), MADDPG and MAPPO. MADDPG and MAPPO learn a separate critic for every agent, which gives the algorithms flexibility to learn different behaviors for agents with different reward functions.

In a mixed or cooperative-competitive setting, environments are neither zero-sum (competitive) nor cooperative, and they do not necessarily need to be general-sum either. A common setting would be environments that require every agent to cooperate with some agents, and compete with others (Busoniu et al., 2008; Canese et al., 2021; Zhang et al., 2021). MADDPG and MAPPO are used here for the same reason as the competitive setting.

### 2.5. Independent algorithms and non-stationarity

One naive approach for applying single-agent RL to the multi-agent setting would be the use of independent learners, where each agent treats every other agent as part of the environment, and learns purely based on individual observations. In a multi-agent setting, the transition probability *P* and reward function *R* are conditioned on the joint action *u*. Since all agents in the environment are learning, their policies constantly change. Therefore, from each independent learner's perspective, the transition probability and reward function appear non-stationary, due to the lack of awareness of other agents' actions. This violates the Markovian property of an MDP, which then causes independent algorithms to be slow to adapt to other agents' changing policies, and as a result, face difficulties in converging to a good policy (He et al., 2016; Hernandez-Leal et al., 2017; Papoudakis et al., 2019).

In this paper, we chose to use a popular off-policy algorithm, Deep Q-Network (DQN) (Mnih et al., 2015), and an on-policy algorithm, Proximal Policy Optimization (PPO) (Schulman et al., 2017). In specific partially observable environments, Deep Recurrent Q-Network (DRQN) (Hausknecht and Stone, 2015) is also utilized. Following the work of Gupta et al. (2017), parameter sharing is utilized for all independent algorithms, such that experiences from all agents are trained simultaneously using a single network. This allows the training to be more efficient (Gupta et al., 2017). The aforementioned independent algorithms are tested in all 3 categories of multi-agent environments.

## 3. Experimental setup

In this section, we introduce the environments used for the experiments, specify the various preprocessing that were applied, and other relevant implementation details.

### 3.1. Environments

The experiments were performed on multiple multi-agent environments from the PettingZoo library (Terry et al., 2020b), which contains the Multi-Agent Particle Environments (MPE) (Lowe et al., 2017; Mordatch and Abbeel, 2017) and multi-agent variants of the Atari 2600 Arcade Learning Environment (ALE) (Bellemare et al., 2013; Terry and Black, 2020).

For the cooperative setting, experiments were performed on a modified version of the 2-player Space Invaders (Bellemare et al., 2013; Terry and Black, 2020), and the Simple Reference MPE environment (Lowe et al., 2017; Mordatch and Abbeel, 2017). In Space Invaders, both agents share the common goal of shooting down all aliens. To make Space Invaders cooperative, we removed the (positive) reward that is given to a player whenever the other player gets hit. Additionally, the environment rewards every agent individually by default. Since a number of cooperative multi-agent algorithms (e.g., QMIX and COMA) assume that only a team reward is given, we modified the reward function such that a team reward is given instead (i.e., both agents receive the sum of their individual rewards). This setup exemplifies the multi-agent credit assignment problem, the effect of which is studied more closely in the Section 4.1.1. On the other hand, in the Simple Reference environment, two agents are rewarded by how close they are to their target landmark. However, the target landmark of an agent is only known by the other agent, as a result communication is required for both agents to navigate successfully to their target landmarks.

For the competitive setting, we performed experiments on three 2-player zero-sum competitive games from the Atari suite—Boxing, Pong and Space War. For the mixed setting, we opted for the Simple Tag and the Simple Adversary MPE environments. Simple Tag is a Predator-Prey environment that consists of 3 predators and a prey (Mordatch and Abbeel, 2017). The prey travels faster and has to avoid colliding with the predators, while the 3 predators travel slower and have to work together to capture the prey. The rewards received by the prey and a predator sum to 0 (i.e., the prey gets a negative reward for collision, while the predators get rewarded positively), and all predators receive the same reward. The prey is also negatively rewarded if it strays away from the predefined area (a 1 × 1 unit square). This environment is general-sum because it contains 3 predators and a single prey. On the other hand, Simple Adversary consists of 2 cooperative agents and an adversary agent. Both cooperative agents receive equal reward, based on the distance of the closest agent to the target and the negative distance of the adversary to the target. The adversary is also rewarded based on its distance to the target, but unlike the cooperative agents, it has to infer the location of the target based on the location of the cooperative agents, which it observes. Therefore, the cooperative agents have to cooperate in order to split up to get close to the target while deceiving the adversary away from the target. Similarly, the Simple Adversary environment is also general-sum.

### 3.2. Preprocessing

For the MPE environments, no preprocessing was done, and default environment-parameters were used for all MPE experiments.

For the Atari environments, following the recommendations of Machado et al. (2018), we performed the following preprocessing:

- Reward clipping to ensure that the rewards at every timestep were clipped between the range of [-1, 1].
- Sticky actions with a probability of 0.25.
- Frame skip of 4.

The number of steps per episode was set to a limit of 200 for all Atari environments, as that yielded the best results in general. Additionally, no-op resets were also performed on the first 130 frames for Space Invaders, and the first 60 frames for Pong.

Furthermore, the action spaces for both Atari environments were shrunk to their effective action spaces in order to improve learning efficiency. For all competitive environments specifically, we also concatenated a one-hot vector of the agent's index to the observations so that independent algorithms can differentiate one from the other when parameter sharing is utilized. The effect of this addition is studied more closely in Section 4.5.

All preprocessing were performed using the SuperSuit library (Terry et al., 2020a).

### 3.3. Implementation

Implementations of all algorithms were based on the following open-sourced libraries/reference implementations:

- Implementation of DQN and DRON were based on the Machin library (Li, 2020).
- Implementation of independent PPO was based on Stable Baselines3 (Raffin et al., 2019).
- Implementation of DRQN, QMIX, COMA, and CommNet came from the MARL-Algorithms GitHub repository (starry sky6688, 2019).
- Implementation of MADDPG came from the original code implementation (Lowe et al., 2017).
- Implementation of MAPPO came from the original code implementation (Yu et al., 2021).

For both DQN and DRON, the underlying DQN implementations included Double DQN (Van Hasselt et al., 2016), the dueling architecture (Wang et al., 2016) and priority experience replay buffer (Schaul et al., 2015). On the other hand, the implementation of DRQN did not use any of the aforementioned add-ons. For PPO and MAPPO, 4 parallel workers were used for all environments with homogeneous state and action spaces. Default hyperparameters were used for all algorithms, and no hyperparameter tuning was performed. Details of the hyperparameters used can be found in the Supplementary material.

All experiments were performed across 5 different seeds. Parameter sharing was utilized for all algorithms throughout the experiments for all environments with homogeneous state and action spaces. For multi-agent algorithms that perform centralized training (e.g., QMIX, COMA, MADDPG etc.), the global states were represented by the concatenation of all agents' local observations. We also used the 128-byte Atari RAM as state inputs, rather than visual observations. This allows the algorithms to focus their learning on control rather than on both control and perception, improving learning efficiency.

For the mixed setting, the observation and action spaces differ between predators and the prey in the Simple Tag environment, while the observation spaces of the cooperative agents differ from the adversary agent in the Simple Adversary environment. As a result, in both environments, none of the agents have their parameters shared. Parameters between the predators/cooperative agents are also not shared to ensure that no bias is introduced (since they would have more data to learn from compared to opposing agents in their respective environments).

## 4. Experimental results and discussion

In this section, we highlight the experiments performed on all environments, and provide discussions about the obtained results. Although we only refer to the plotted figures in the following subsections for performance comparison purposes, the final score obtained by every algorithm in all environments are also reported in Tables 1–3.

**Table 1:** Final scores (mean and standard deviation) of algorithms obtained over the last 100 episodes across all 5 seeds in the Space Invaders and Simple Reference environments.

**Algorithm**	**Simple reference**	**Algorithm**	**Space invaders**
QMIX	–46.2 ± 28.4	QMIX	12.5 ± 3.28
RMAPPO	**–36.8 ± 11.8**	RMAPPO	16.2 ± 3.31
MAPPO	–38.0 ± 14.0	MAPPO	**22.5 ± 3.45**
CommNet	–58.2 ± 18.5	MADDPG	9.78 ± 0.98
COMA	–36.6 ± 13.3	DRQN	12.2 ± 1.61
DRQN	–52.8 ± 19.1	DQN	15.7 ± 3.70
DQN	–66.0 ± 33.0	PPO	19.8 ± 3.52
PPO			7.92 ± 2.69

The highest score in each environment is bolded.

**Table 2:** Overall winrate percentage of various algorithms across Boxing, Pong and Space War environments.

**Algorithm**	**Boxing(%)**	**Pong(%)**	**Space war(%)**
DQN	**94**	88	70
PPO	68	36	52
DRON	84	**90**	68
MADDPG	54	28	72
MAPPO	64	40	66
RMAPPO	64	24	**76**

The highest winrate percentage for every environment is bolded.

**Table 3:** Final scores (mean and standard deviation) of algorithms obtained over the last 100 episodes across all 5 seeds in the Simple Tag and Simple Adversary environments.

	**Simple Tag**	
**Algorithm**	**Predator**	**Prey**
DQN	3.24 ± 7.99	**−4.93 ± 9.36**
PPO	2.46 ± 7.60	−47.0 ± 57.9
MADDPG	4.40 ± 9.24	−8.10 ± 12.2
RMAPPO	**14.0 ± 20.5**	−16.4 ± 21.2
MAPPO	13.4 ± 19.1	−20.8 ± 22.5
	**Simple Adversary**	
**Algorithm**	**Adversary**	**Cooperative Agent**
DQN	−18.2 ± 9.27	7.56 ± 7.85
PPO	−52.7 ± 21.7	**34.2 ± 20.0**
MADDPG	**−15.6 ± 7.65**	7.24 ± 6.35
RMAPPO	−29.4 ± 15.0	10.8 ± 16.4
MAPPO	−24.9 ± 12.2	9.11 ± 13.8

The highest scores for every type of agent in both environments are bolded.

### 4.1. Cooperative

**Figure 1:**
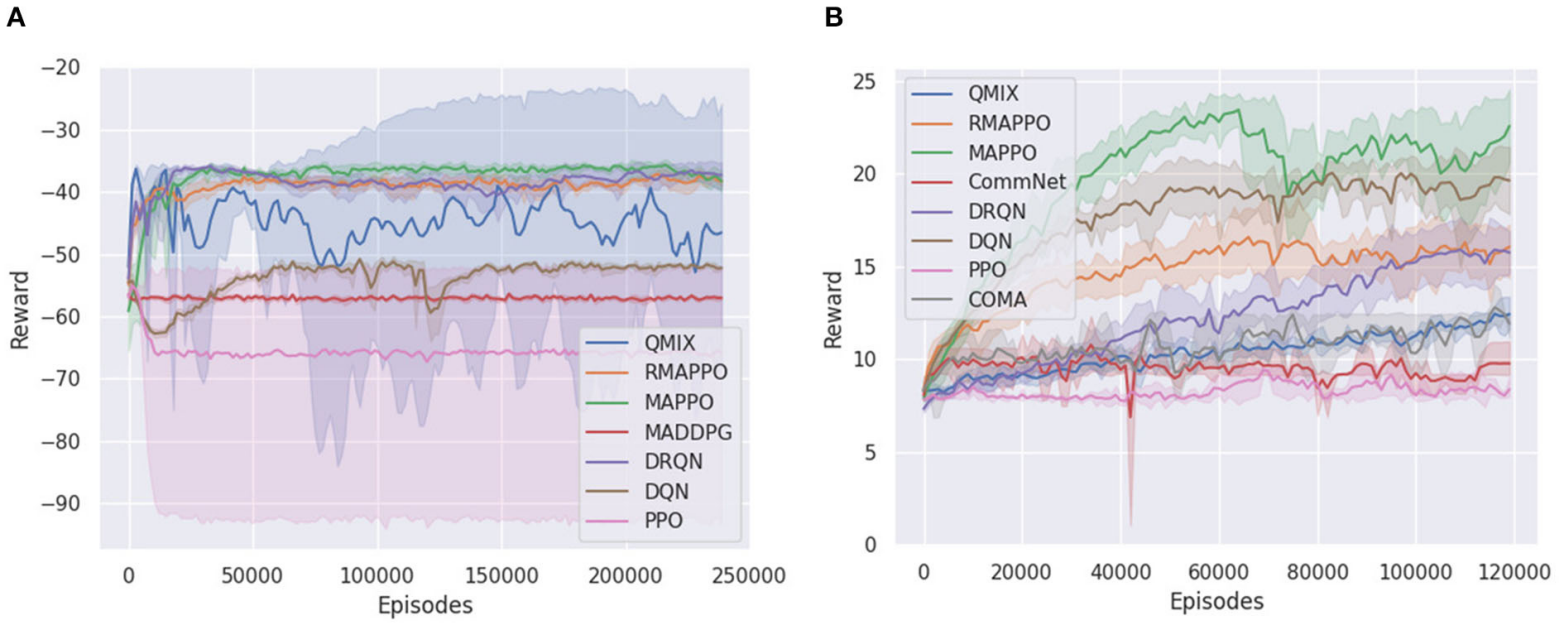
Training curves of various algorithms in two cooperative environments. For every algorithm, the solid line represents the mean reward per episode, while the shaded region represents the 95% confidence interval around the mean. **(A)** Shows training curve for Simple Reference environment, **(B)** shows training curve for Space Invaders environment.

We ran the various algorithms on the Simple Reference environment for 240k episodes (6 × 10^6^ steps). From Figure 1A, it could be observed that all independent algorithms converged to a lower score, except for DRQN, whose recurrence allowed it to vastly outperform DQN and converge to a score on par with multi-agent algorithms. However, this trend was not observed when comparing MAPPO to its recurrent variant (i.e., RMAPPO), as MAPPO performs equally well as RMAPPO. We hypothesize that since MAPPO's centralized critic learns based on the joint observation and action of both agents, this minimizes the amount of partial observability of every agent, and allows each agent to learn to communicate with other agents effectively without recurrence. In contrast, for independent algorithms, such as DQN, where the interactions between the agents are not explicitly learned (since all other agents are treated as part of the environment), adding recurrence could help mitigate some resulting partial observability, hence improving their performance, as described above.

Unlike the Simple Reference environment, the Space Invaders environment seemed to favor non-recurrent variants of algorithms (Figure 1B). MAPPO vastly outperformed RMAPPO, and similarly DQN outperformed DRQN. This is also likely the underlying reasoning behind the comparatively poorer performance of the multi-agent algorithms, such as QMIX, COMA and CommNet, all of which were implemented with recurrent neural networks under the CTDE scheme.

Additionally, since there is no unit collision in the Space Invaders environment (i.e., agents can move past each other without being blocked), they do not have to coordinate between themselves to achieve a high score in the environment; a good policy can be learned solely by having agents maximize their individual rewards. This explains the strong performance that was achieved by DQN. Also, since this is a cooperative task with both agents having identical goals, learning separate representations for individual agents is not very important; the learning of both agents assist each other. This is shown in **Figure 8B** in Section 4.5, where the addition of an agent indicator did not yield any performance improvement for DQN on Space Invaders.

Given such circumstances, it is interesting to observe the stronger performance of MAPPO compared to the independent algorithms. By conditioning on the joint action, MAPPO's critic has full observability into the joint action that resulted in the team reward. Therefore, the observed reward is unbiased, which allows the learning process to be more efficient. In contrast, independent algorithms have to learn from a noisy team reward signal, where an agent could receive a large positive team reward even when it did nothing. This relates to the problem of credit assignment in MARL, noted in prior works (Hernandez-Leal et al., 2019).

#### 4.1.1. Multi-agent credit assignment problem in fully observable settings

In this section, we attempt to study the effect of using a team reward signal, rather than individual reward signals on various independent and multi-agent algorithms in a fully observable environment. When team rewards are the only rewards given, these reward signals are noisy for independent algorithms because the agent, which treats every other agent as part of the environment, does not know the actions taken by other agents. This makes it difficult for independent algorithms' agents to learn how their individual actions contribute to the team reward signal. We performed the experiments on Space Invaders, in which the default agents receive individual rewards from the environment. To study the effect of the multi-agent credit assignment problem, we performed two runs per algorithm, one with team rewards only, and the other with individual rewards only (i.e., agents are rewarded independently by the environment).

**Figure 2:**
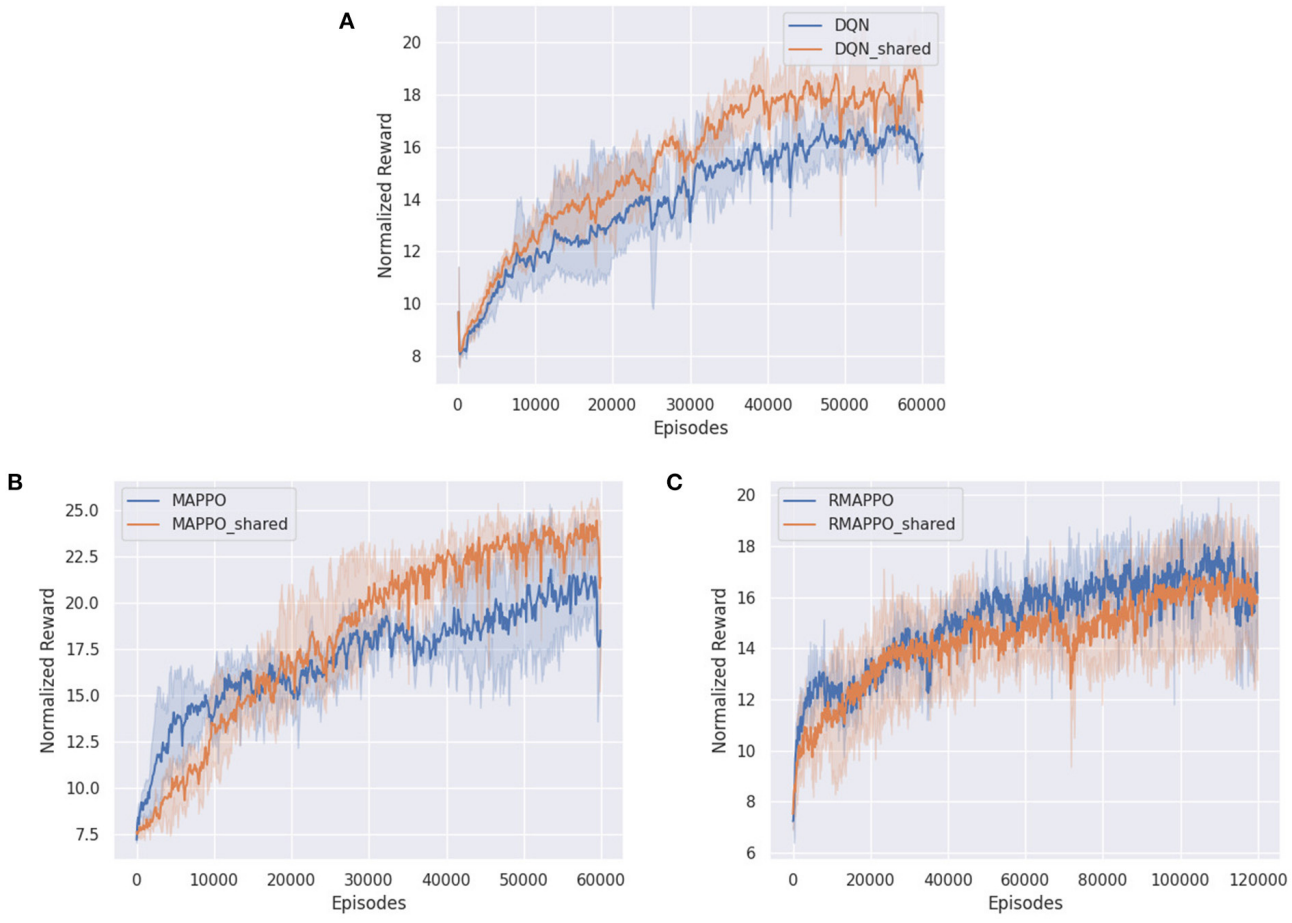
Training curves of various algorithms in Space Invaders, comparing when individual rewards are given (blue) to when team rewards are given (orange). **(A)** Shows training curve of DQN, **(B)** shows training curve of MAPPO, **(C)** shows training curve of RMAPPO.

For multi-agent algorithms, such as MAPPO (Figure 2B) and RMAPPO (Figure 2C), having a team reward does not have a large effect on the performance of the algorithms. This is expected because these algorithms have critics that learn from the joint action, which allow them to implicitly learn the estimated contribution of every agent without factorization.

On similar lines, regarding independent algorithms, we observe that having team rewards instead of individual ones do not impact their performance adversely (Figure 2A). A plausible explanation could be that since parameter sharing is utilized and all agents receive the same reward for a given joint action, this allows the independent algorithms to correlate actions from different agents that produced similar (high) rewards.

### 4.2. Competitive

**Figure 3:**
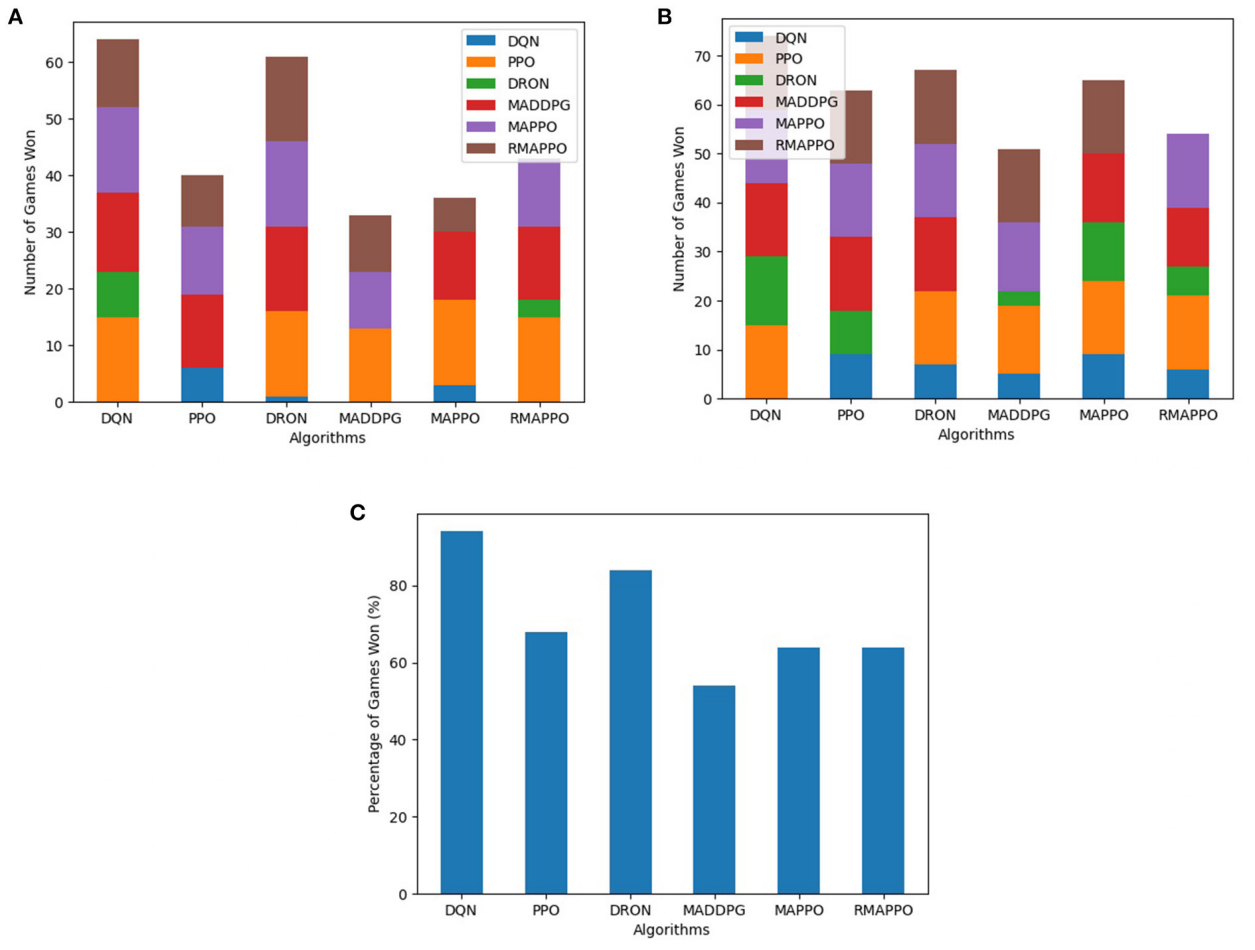
Performance of various algorithms when playing against other algorithms in the Boxing environment. **(A)** Shows the number of games won as the first player, **(B)** shows the number of games won as the second player, **(C)** shows the overall win rate percentage.

**Figure 4:**
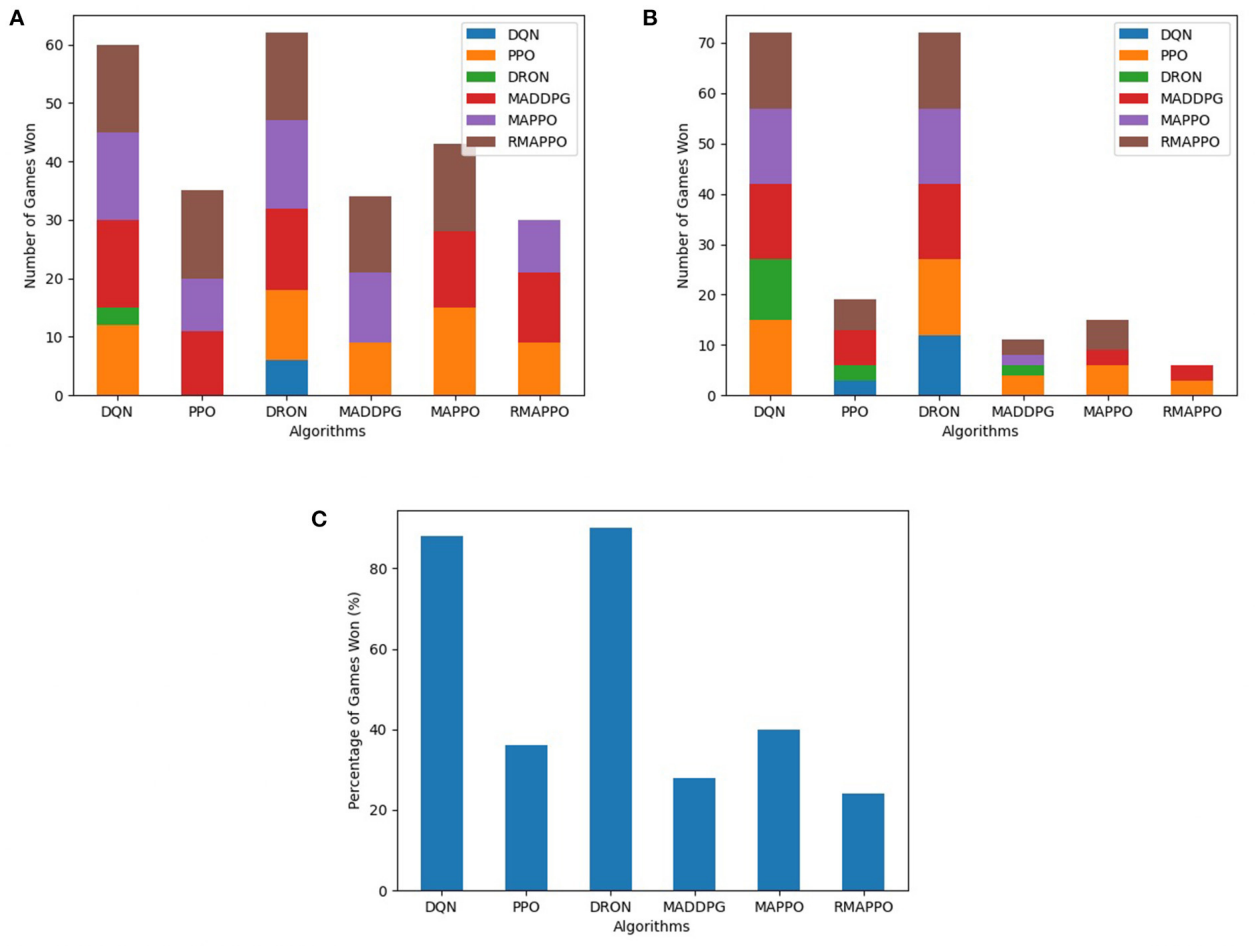
Performance of various algorithms when playing against other algorithms in Pong. **(A)** Shows the number of games won as the first player, **(B)** shows the number of games won as the second player, **(C)** shows the overall win rate percentage.

**Figure 5:**
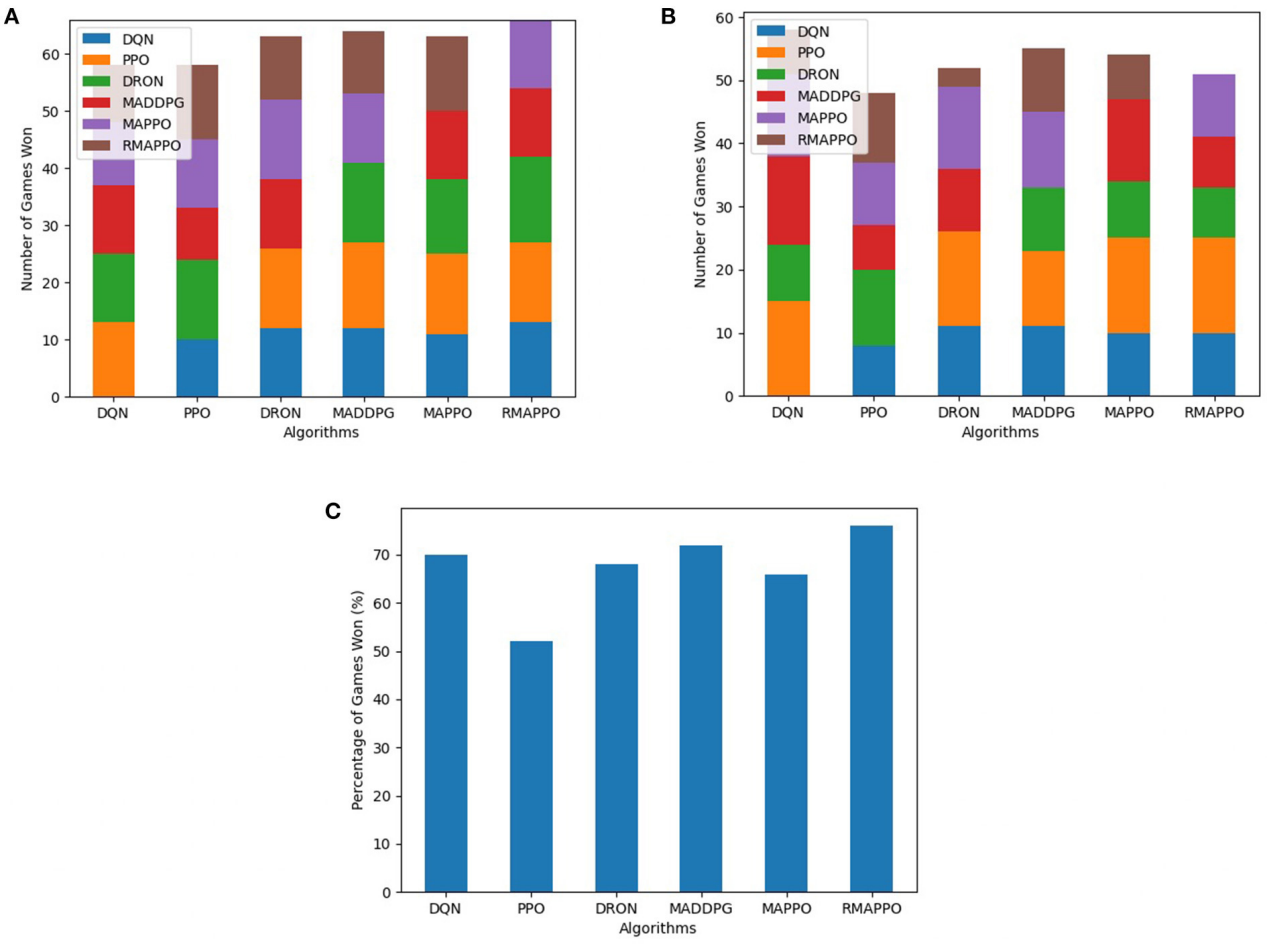
Performance of various algorithms when playing against other algorithms in the Space War environment. **(A)** Shows the number of games won as the first player, **(B)** shows the number of games won as the second player, **(C)** shows the overall win rate percentage.

For every competitive environment, all algorithms were trained for a fixed number of steps (1.2 × 10^7^). Performance evaluation is performed by pitting algorithms head-to-head against each other for 3 episodes for all possible permutations, during which no training is performed. To ensure fairness, the ordering of algorithms (i.e., Algorithm A playing as player 1 or 2) are also taken into consideration. This is because in environments such as Pong, the right paddle player is always the serving player, thus having an advantage. At the end of an episode, the agent of an algorithm that has achieved higher cumulative reward is considered the winner. If both agents achieved the same amount of cumulative reward (draw scenario), both agents are considered to win. The entire evaluation process is repeated across all 5 seeds, and is the basis behind the stacked bar charts (Figures 3–**5**). As previously mentioned, the final winrates percentage obtained by each algorithm across all competitive environments are also reported in Table 2.

Among all algorithms, DQN and DRON are the best performers in Boxing and Pong environment, by a large margin. DQN outperforms DRON in the Boxing environment (Figure 3), while DRON outperforms DQN in the Pong environment (Figure 4). Since both of these environments are reactive in nature, this meant that an agent can learn a good policy solely by understanding how to react to the situation at hand. For instance, in Pong, this meant learning to position the paddle according to the trajectory of the ball (toward the agent). While learning on the joint action could allow agents to learn to better predict the incoming trajectory of the ball, it can be observed that the additional layer of complexity causes the sample efficiency to decrease and only yields diminishing returns. Additionally, since both of these environments are fully observable, critics that learn based on the joint observation of both agents do not necessarily provide any new information.

In the Space Wars environment, two agents shoot missiles at each other. Critically, since the missiles travel faster than the agents and the missiles can ricochet off walls, prediction and positioning are key for shooting and dodging effectively. This makes the environment challenging. In this environment, RMAPPO performed the best, closely followed by MADDPG and DQN (Figure 5). By conditioning on past trajectories, RMAPPO was able to better learn the mechanics of the environment (such as ricocheting missiles), allowing it to have better aim than other algorithms. However, this only yields marginal improvement over the MADDPG and DQN. Even with the complexity of the environment, DQN's performance was nearly on par with MADDPG, and performed better than the rest of the multi-agent algorithms, including DRON and MAPPO.

### 4.3. Mixed

**Figure 6:**
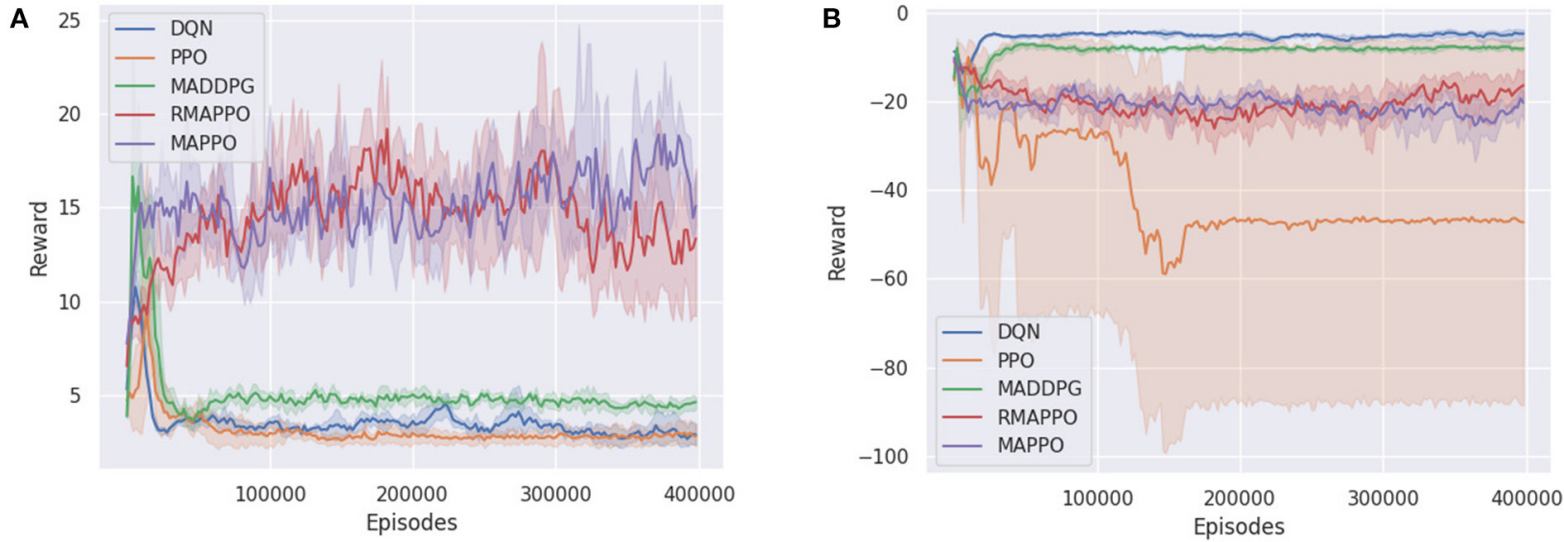
Training curves of various algorithms in the Simple Tag, a Predator-Prey environment. **(A)** Shows the reward of a predator (all predators obtain the same reward), **(B)** shows the reward of the prey.

In the Simple Tag (i.e., Predator-Prey) environment, DQN's prey successfully learned to minimize the number of collisions with the predators, which can be observed by the strong performance achieved by the prey (Figure 6B). However, similar to PPO, since the predators were trained completely independently (i.e., their parameters were not shared), they did not manage to learn how to cooperate with one another to capture the prey (Figure 6A). It is interesting to observe that MADDPG converged to a policy similar to DQN, with the difference being that its predators have learned to cooperate better, thus getting slightly higher rewards compared to DQN's predators (Figure 6A). Subsequently, as a result of the higher rewards obtained by the predators, MADDPG achieves a slightly lower score for its prey (Figure 6B).

MAPPO and RMAPPO, on the other hand, learned a different strategy. As we can observe from the comparatively noisier curves obtained from their predators and preys (Figures 6A,B), there is a constant tug-of-war between the prey and the predators—as the predators learn how to cooperate better, their scores increase, which subsequently causes the prey to learn how to dodge, decreasing the predators' scores, and vice versa. Since the predators of MAPPO and RMAPPO achieves a much higher score compared to all other algorithms, this is indicative that the predators have successfully learned to cooperate to trap the prey.

**Figure 7:**
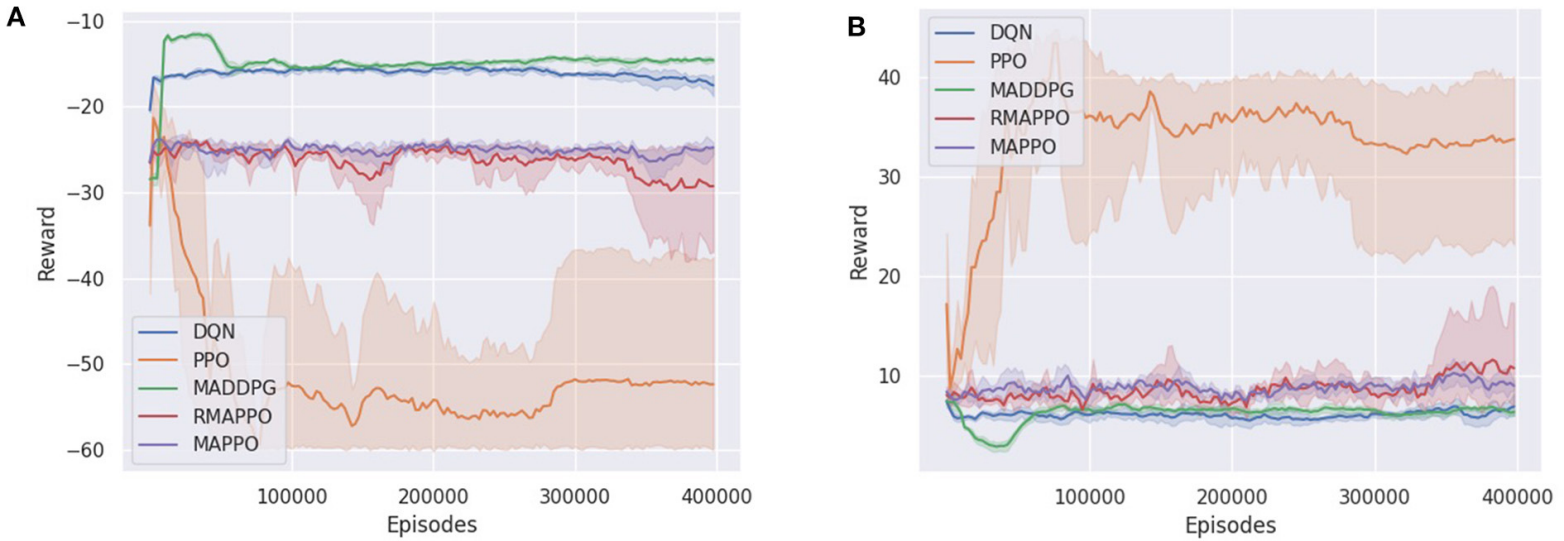
Training curves of various algorithms in the Simple Adversary environment. **(A)** Shows the reward of the adversary, **(B)** shows the reward of a cooperative agent (both cooperative agents obtain the same reward).

Similar to the Simple Tag environment, the Simple Adversary environment exhibits similar patterns in terms of relative performances of the various algorithms. DQN and MADDPG converged to a similar policy, where the stronger performance of the adversary agent implies that the adversary agent was able to better locate the target location (Figure 7A). In other words, the two cooperative agents were less successful at deceiving the adversary agent into an incorrect target. On the other hand, MAPPO and RMAPPO's adversary agent converged to a substantially lower score (Figure 7A), but conversely the cooperative agents were able to achieve greater score than those of DQN and MADDPG (Figure 7B). This is indicative that the cooperative agents (of MAPPO and RMAPPO) were more successful in learning to cooperate by positioning close to the true target while deceiving the adversary away from it.

### 4.4. Performance of independent PPO

Throughout all experiments performed, PPO exhibits poor performance in most of the environments across all 3 settings. While PPO has been shown to work well in a wide variety of environments (Schulman et al., 2017; OpenAI et al., 2018; Berner et al., 2019; Yu et al., 2021), PPO is highly sensitive to the choice of implementation and hyperparameters, as reported in prior works (Andrychowicz et al., 2020; Engstrom et al., 2020). We find that this problem is exacerbated in multi-agent environments, especially in the mixed settings, since those are the hardest.

### 4.5. Importance of agent indicator

In this section, we list some interesting findings from the addition of agent indicators to independent algorithms when utilizing parameter sharing.

**Figure 8:**
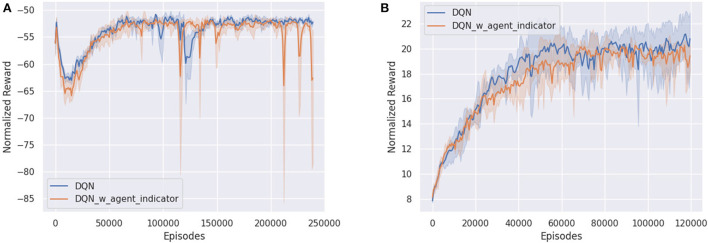
Comparing DQN with (blue) and without (orange) agent indicators in **(A)** Simple Reference and **(B)** Space Invaders environment.

Interestingly, in both cooperative environments, there was no noticeable improvement in the performance of DQN when an agent indicator was added (Figures 8A,B). As was previously discussed, in the case of Space Invaders, since both agents have identical goals and similar representations, there is little need to distinguish between either agent. On the other hand, due to the partially observable nature of the Simple Reference environment, DQN performed similarly poorly, regardless of whether agent indicators were present. In this case, the addition of recurrence would have resulted in a much more significant difference instead, as was previously shown.

**Figure 9:**
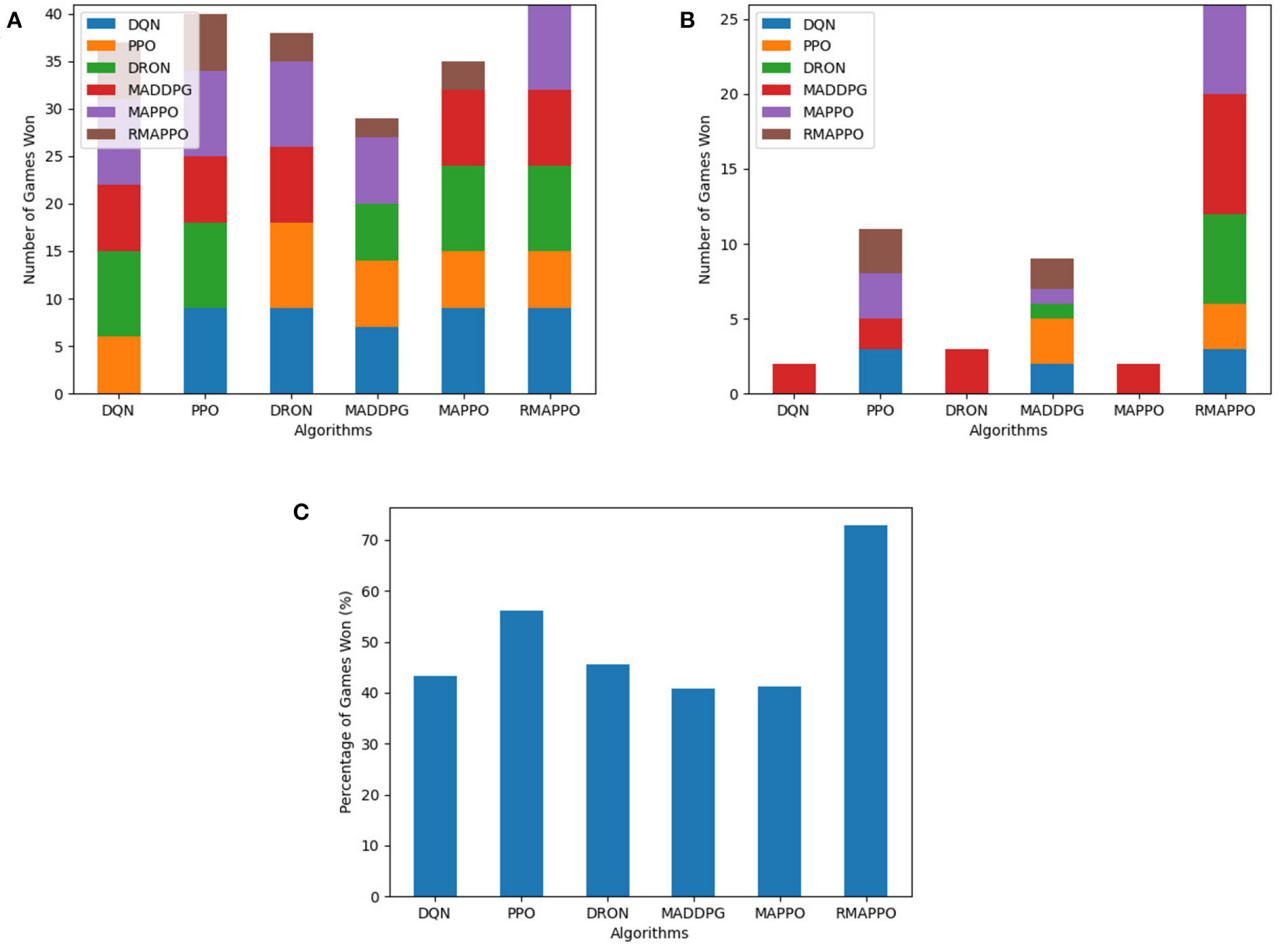
Performance of various algorithms when playing against other algorithms in Pong without agent indicators across 3 seeds. **(A)** Shows the number of games won as the first player, **(B)** shows the number of games won as the second player, **(C)** shows the overall win rate percentage.

Conversely, for the Pong environment, even though it is also fully observable (akin to Space Invaders), the representation of both agents are not interchangeable. Utilizing parameter sharing without agent indicators, all algorithms struggled to learn due to the inability to tell which paddle were they controlling at every timestep. The only exception was RMAPPO (Figure 9), which was able to condition on the sequence of previous observations and actions to infer which paddle was it controlling.

## 5. Conclusion

In this section, we provide a summary of the findings and discussions from the previous sections.

### 5.1. Cooperative

In the cooperative setting, for environments where individual agents have full observability such as Space Invaders, we showed that independent algorithms can perform even better than certain multi-agent algorithms. Furthermore, we showed that independent algorithms are able to cope well with the multi-agent credit assignment problem in environments that are fully observable with a relatively small number of agents, and where every agent has the same task. On the other hand, in the Simple Reference environment where the need for agents to communicate induces partial observability, adding recurrence allowed independent algorithms to perform as well as other multi-agent algorithms. We also discussed the significance of learning on the joint observation and action, rather than individual ones, and showed that MAPPO performs as well as DRQN in the Simple Reference environment, without the need for an RNN. Moreover, in Space Invaders, MAPPO was able to consistently achieve the highest score amongst all other algorithms.

### 5.2. Competitive

In the Boxing and Pong environment, DRON and DQN were able to outperform all other algorithms. We argued that this is due to the reactive nature of both environment, which results in diminishing returns for multi-agent algorithms that learn joint actions. On the other hand, in the more complex Space Wars environment, RMAPPO performed the best as it was able to leverage on past observations to make better predictions. However, DQN still performed on par or better than other multi-agent algorithms. Furthermore, we showed that with the addition of agent indicators, independent algorithms were able to learn robust policies using parameter sharing in Pong.

### 5.3. Mixed

In both mixed environments, we saw that since there were no parameter sharing to induce cooperation, cooperative agents of independent algorithms were unable to learn how to cooperate with each other to compete with the opposing agent. This was reflected in DQN's stronger performance as the prey in the Simple Tag environment, and the adversary in the Simple Adversary environment. Agents of MAPPO and RMAPPO, on the other hand, were able to learn to cooperate, leading to higher rewards as predators in Simple Tag, and as cooperative agents in Simple Adversary. Furthermore, the noisiness of graphs suggest that there is a constant tug-of-war between both opposing parties, as one tries to outsmart the other. Interestingly, in both mixed environments, MADDPG exhibits similar characteristics to DQN, suggesting that its cooperative agents in both environments also faced difficulties in learning to cooperative.

## 6. Future work

In this section, we highlight some future work that could potentially bring more insights into having a broader understanding of dealing with non-stationarity and partial observability for independent algorithms, both of which are common in the multi-agent setting. In the Space Invaders environment, we observed that independent algorithms were able to learn well with just a team reward. Future work could be done to determine if this was only the case for fully observable environments, or under what conditions would independent algorithms still be able to cope with the multi-agent credit assignment problem. It would also be interesting to study the performance of non-recurrent variants of multi-agent algorithms such as QMIX and COMA in fully observable environments. Since the experiments performed in this paper only included fully-observable competitive and mixed environments, future work can also include a more diverse set of environments, such as partially observable competitive and mixed environments.

## Data availability statement

The original contributions presented in the study are included in the article/Supplementary material, further inquiries can be directed to the corresponding author/s.

## Author contributions

KL performed the experiments and analyzed the results. SG assisted in designing the experiments and analyzing the results. MC provided access to computing resources and gave numerous feedback throughout the entire process. All authors contributed to the article and approved the submitted version.

## Funding

This project was made possible through grants from the Canada Wildfire Strategic Network and the Discovery Grant Program from the National Research Council of Canada (NSERC).

## Conflict of interest

The authors declare that the research was conducted in the absence of any commercial or financial relationships that could be construed as a potential conflict of interest.

## Publisher's note

All claims expressed in this article are solely those of the authors and do not necessarily represent those of their affiliated organizations, or those of the publisher, the editors and the reviewers. Any product that may be evaluated in this article, or claim that may be made by its manufacturer, is not guaranteed or endorsed by the publisher.
