# Supplementary material for: Investigation of independent reinforcement learning algorithms in multi-agent environments
Source: Front Artif Intell. 2022 Sep 20;5:805823. doi: 10.3389/frai.2022.805823 (PMC9530713; doi:10.3389/frai.2022.805823)
Supplement: Supplementary file 1 [file Data_Sheet_1.PDF]

Supplementary Material

1 HYPERPARAMETERS

In this section, we specify the hyperparameters used for all algorithms throughout the experiments.

Hyperparameter	Value
fully-connected layer dimensions	512×256
optimizer	Adam
learning rate	0.001
discount factor	0.99
replay buffer size	1×10^6
batch size	256
loss function	MSE
initial epsilon	1
epsilon decay rate	0.9999
double	True
dueling	True
priority	True

Table S1. Hyperparameters for DQN and DRON

Hyperparameter	Value
fully-connected layer dimensions	64×64
number of environments	4
optimizer	Adam
number of steps	episode length
number of epochs	10
minibatch size	episode length*# of agents*4
discount factor	0.99
GAE lambda	0.95
learning rate	0.0007
value function coefficient	0.5
clip	0.2
entropy	0.01

Table S2. Hyperparameters for PPO

Hyperparameter	Value
fully-connected layer dimensions	64×64
optimizer	Adam
learning rate	0.01
discount factor	0.95
replay buffer size	1×10^6
batch size	1024
critic loss function	MSE
gradient clip norm	0.5

Table S3. Hyperparameters for MADDPG

Hyperparameter	Value
fully-connected layer dimensions	64×64
number of environments	4
optimizer	Adam
number of epochs	10
minibatch size	1600
discount factor	0.99
GAE lambda	0.95
learning rate	0.0007
value function coefficient	0.5
clip	0.2
entropy	0.01
RMAPPO-specific Hyperparameters	
number of GRU layers	1
hidden state dimension	64

Table S4. Hyperparameters for MAPPO and RMAPPO

Hyperparameter	Value
discount factor	0.99
optimizer	RMSProp
number of GRU layers	1
hidden state dimension	64
gradient clip norm	10
batch size	256
COMA-specific Hyperparameters	
critic (fully-connected) dimension	128
actor learning rate	0.0004
critic learning rate	0.003
QMIX/DRQN-specific Hyperparameters	
hypernetwork dimension	64
learning rate	0.0005
epsilon	linear decay from 1 to 0.05
buffer size	1×10^6

Table S5. Hyperparameters for COMA, QMIX, DRQN and CommNet
